# Antioxidant Activity and Preclinical Safety of *Semen persicae* Extract

**DOI:** 10.3390/ijms25168580

**Published:** 2024-08-06

**Authors:** Jing Yang, Yu Liu, Yingying Song, Qinqin Liu, Liqiong Jin, Ruofeng Shang

**Affiliations:** 1 Gansu Analysis and Research Center, Lanzhou 730000, China; phoenixdudu@126.com (J.Y.); 13369454695@163.com (Y.L.); songyingying1988@126.com (Y.S.); 2 Key Laboratory of New Animal Drug Project, Gansu Province/Key Laboratory of Veterinary Pharmaceutical Development, Ministry of Agriculture and Rural Affairs/Lanzhou Institute of Husbandry and Pharmaceutical Sciences of CAAS, Lanzhou 730050, China; l18736127157@163.com

**Keywords:** semen persicae extract, antioxidant activity, cytotoxicity, acute toxicity, sub-acute toxicity

## Abstract

*Semen persicae* is the dried mature seeds of *Prunus persica* (L.) Batsch and *P. davidiana* (Carr.) Franch and is commonly used in traditional Chinese medicine (TCM) formulations because of its variety of biological effects. The present study aimed to evaluate the antioxidant activity and toxicity profiles of semen persicae extract (SPE) after determining the amygdalin content (4.95%) using HPLC. Regarding the in vitro antioxidant activity, SPE with 2 mg/mL concentration scavenged 1,1-diphenyl-2-picrylhydrazyl (DPPH), hydroxyl, and ABTS free radicals with rates of 51.78%, 55.47%, and 57.16%, respectively. The same concentration of SPE chelated 30.76% Fe^2+^. The in vitro cytotoxicity study revealed that SPE induced 92.45% cell viabilities of HEPG2 even at 2000 μg/mL. In the acute toxicity study, oral administration of SPE did not provoke mortality or any toxic signs at doses up to 2000 mg/kg bw. Repeated oral administration for 28 days at doses of 100, 300, and 600 mg/kg per day in rats did not show any toxicity signs or gross pathological abnormalities. The results of the present research provide basic reference data for SPE with a moderate effect on antioxidant activity and low toxicity for future screening of biological and pharmacological properties.

## 1. Introduction

The generation of reactive oxygen species (ROS) is associated with some factors, such as inflammation, normal biochemical reactions, and higher levels of dietary xenobiotics [1,2]. Excessive production of ROS leads to the condition of oxidative stress, which can be effectively neutralized by enhancing cellular defenses in the form of antioxidants [2,3]. Additionally, appropriate supplementation with exogenous antioxidants may help reduce oxidant damage caused by ROS [4]. In recent years, natural antioxidants have been demonstrated to be effective for reducing this oxidant damage by removing free radicals from the body with few side effects [5,6].

Semen persicae, a dry mature seed cultivated in the fruits of *Prunus persica* (L.) Batsch and *P. davidiana* (Carr.) Franch, is commonly used in traditional Chinese medicine (TCM) formulations [7]. Modern pharmacological studies have proved that the main chemical constituents of semen persicae are liposome, cyanogenic glycosides, flavonoids, saccharides, essential oil, proteins, and amino acids, in which liposome is the most abundant chemical composition, especially triacyl glycerol (88.6–92.1%), 1,2-biacyl glycerol (2.2–2.3%), and sterol ester (1.0–1.6%) [8,9]. These active ingredients endow semen persicae with a wide range of biological effects, including accelerating blood circulation [10,11], mitigating poststroke disorders [12,13], inhibiting platelet aggregation, and exerting anti-tumor activity [11,14].

Amygdalin can be found in many plant families, with varying contents. In peach seeds (*Prunus persica* (L.) Batsch), its content has been reported as 6.81 ± 0.02 mg/g [15]. Amygdalin is one of the most important active ingredients of semen persicae, with many pharmacological effects, including anti-inflammatory [16], analgesic [17], antinociceptive [18], and anticancer activity [19]. Therefore, the content of amygdalin has been used as a chemical indicator in quality control of the crude drug [20].

After clinical application for thousands of years, the therapeutic effects of semen persicae are certainly positive [21]. Unfortunately, the improper usage or overdosage of semen persicae taken orally result in slightly poisonous effects [21,22]. This may be related to amygdalin, which can be hydrolyzed into prunasin and ultimately decomposed into benzaldehyde and hydrogen cyanide (HCN) under the β-glucosidase action of the gut flora in the proximal part of the jejunum [20,23,24,25]. However, the information about the evaluation on antioxidant activity and toxicity of semen persicae extract (SPE) is limited. On the other hand, the chemical compositions of SPE have some discrepancies when different extraction processes or producing areas of semen persicae are used, which may result in diversity in its biological activity and toxicity. In this regard, the present study aimed to investigate the effects of SPE extracted by our process on antioxidant activity and its acute and subacute (28 days) toxicity using in vivo experimental models.

## 2. Results

### 2.1. Phytochemical Characterization

**Figure 1:**
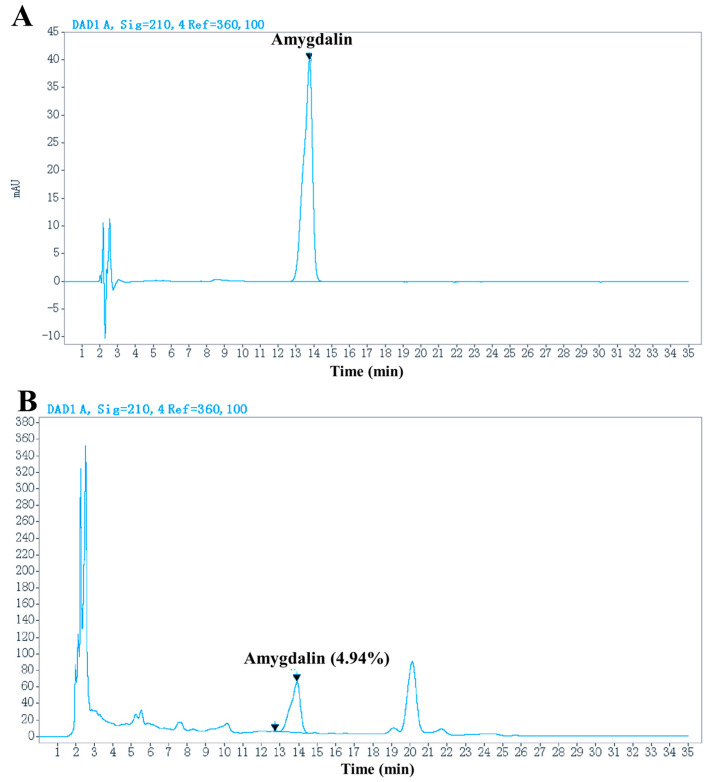
HPLC chromatogram of the standard amygdalin (**A**) and that in extract (**B**).

The percentage yield of ethanolic extract of semen persicae was 4.78% with light yellowish amorphous powder. The extract contained 4.94% amygdalin (Rt = 13.76 min), as analyzed by HPLC (Figure 1).

### 2.2. DPPH Free Radical Scavenging Activity

**Figure 2:**
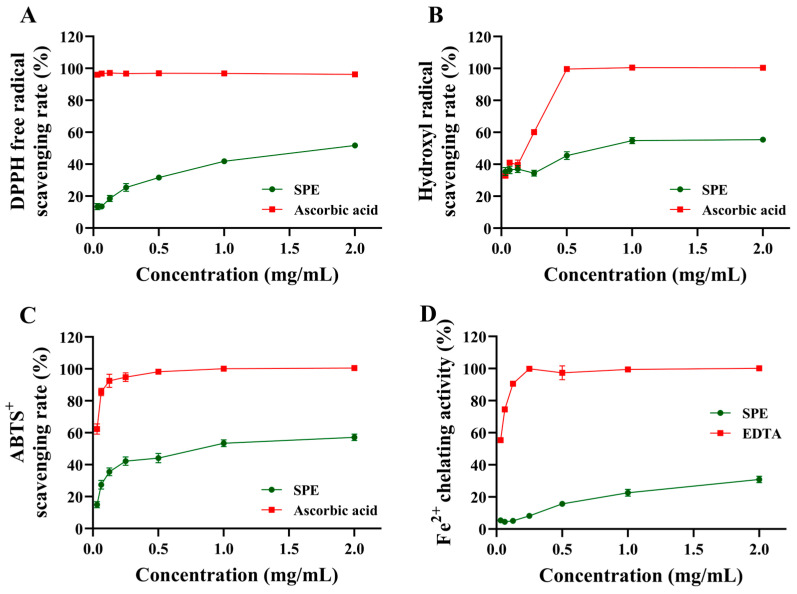
Antioxidant activity of SPE. (**A**) DPPH radical scavenging activity, (**B**) hydroxyl radical scavenging activity, (**C**) ABTS^+^ scavenging activity, (**D**) Fe^2+^-chelating activity. Data are the means of three replicates with standard deviations.

The proton scavenging activity of SPE was determined using DPPH, and the results are shown in Figure 2A. The scavenging rate of SPE correlated well with the concentration, with a concentration-dependent mode demonstrating its clear scavenging activity. In particular, the highest concentration (2 mg/mL) of SPE scavenged DPPH with a rate of up to 51.78 ± 0.42%. In the tested concentrations, the IC_50_ value for SPE was estimated to be 2.05 mg/mL. However, ascorbic acid displayed potent activity for scavenging DPPH free radicals compared to that of SPE. Even at the lowest concentrations (0.03125 mg/mL), ascorbic acid was found to scavenge 96.75% of DPPH radicals.

### 2.3. Hydroxyl Radical Scavenging Activity

The hydroxyl radical scavenging assessment is usually employed to evaluate the antioxidant activity of a natural product. Resembling DPPH radical scavenging effects, the hydroxyl radical scavenging rate (%) of SPE showed a concentration-dependent mode, but with a minor increasing extent from 35.74–55.47% (Figure 2B). The IC_50_ value was tested to be 0.99 mg/mL, indicating the moderate capacity of SPE for scavenging hydroxyl radicals. Again, ascorbic acid displayed a higher activity than that of SPE to scavenge hydroxyl radicals. In particular, 0.5 mg/mL of ascorbic acid reached a 100% hydroxyl radical scavenging rate.

### 2.4. ABTS^+^ Scavenging Activity

The values of SPE for scavenging ABTS^+^ ranged from 14.92% to 57.16% with a concentration-dependent mode, which resembled the scavenging effects on DPPH and hydroxyl radicals (Figure 2C). In this assay, the IC_50_ value was tested to be 0.75 mg/mL, reflecting the moderate scavenging capacity of SPE for ABTS^+^. Ascorbic acid still possessed the highest scavenging capacity for ABTS^+^, with an IC_50_ value of 2.54.

### 2.5. Fe^2+^-Chelating Assay

The Fe^2+^-chelating capacity of SPE was assessed by measuring iron–ferrozine complexes to further estimate its antioxidant activity, as shown in Figure 2D. The obtained results demonstrated the Fe^2+^-chelating effect of SPE in concentration-dependent mode, with activity from 5.19 to 30.76%. In the present Fe^2+^-chelating capacity study, it was found that the IC_50_ value was estimated to be 9.95 mg/mL, demonstrating the low chelating activity of SPE. Conversely, EDTA demonstrated an excellent ability to chelate Fe^2+^. More specifically, EDTA was found to chelate Fe^2+^ even at a 0.25 mg/mL concentration.

### 2.6. Cytotoxicity Evaluation

**Figure 3:**
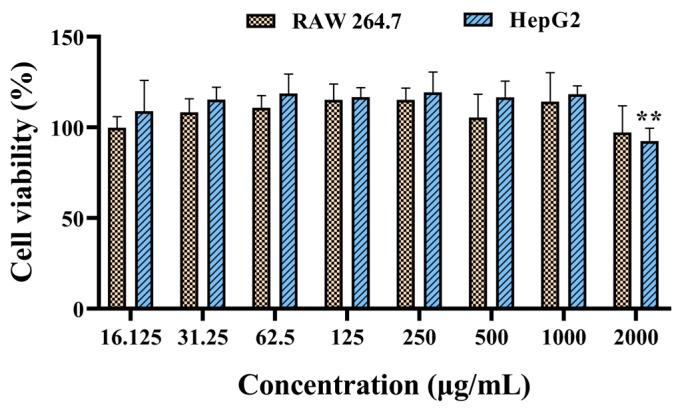
In vitro toxicity profiles of SPE measured using CCK-8 method. The values represent the mean and standard deviation from three independent experiments. ** *p* < 0.01 versus the other groups.

Using RAW 264.7 and HepG2 cells, preliminary cytotoxicity studies of SPE were performed through dose–response studies (Figure 3). The results revealed that SPE only induced negligible cytotoxicity on RAW 264.7 up to 2000 μg/mL, with no significant difference when compared to that in other treatment groups. The cell viability of HepG2 induced by SPE at 2000 μg/mL was 92.45%, displaying a significant difference when compared to that in the negative control group. However, no significant differences were observed among the groups treated with ≤1000 μg/mL of SPE.

### 2.7. Acute Oral Toxicity

After oral administration, one single 2000 mg/kg dose of the vehicle or SPE in female rats did not produce any signs of toxicity at the first or second round. All the treated animals survived until programmed euthanasia. The gain in body weight did not show any significant alterations in SPE-treated rats when compared with the vehicle control group after 7 and 14 days (Supplementary Information Table S1). No pathological signs in the morphology of the organs were observed in the gross necropsies of any of the rats. The absolute and relative organ weights of the heart, liver, spleen, lung, kidney, uterus, and ovaries showed no significant change (*p* > 0.05) in SPE-treated rats when compared to those in the control group (Table 1).

### 2.8. Subchronic Toxicity Study

After repeated oral doses of SPE for 28 days, no death or obvious abnormal or toxic signs were observed during the study period in any animals in either the treatment or control groups. Furthermore, no noticeable differences were observed in food intake (Supplementary Information Figure S1) or treatment-related changes in body weight among the four groups (Supplementary Information Figure S2).

The effects of SPE on the hematological parameters and serum biochemical effects are summarized in Table 2 and Table 3. There were no significant changes in the hematology results in the treatment groups, except for the mean platelet volume (MPV), which showed a significant increase (*p* < 0.05 or *p* < 0.01) in rats of both sexes which received 300 or 600 mg/kg of SPE. With biochemical parameters, a significant change (*p* < 0.05) in the levels of creatinine in female rats of the 300 mg/kg group was observed when compared to the control group.

At the time of necropsy, the histopathological findings of the organs showed no alterations in color or texture when compared to the control group. The organ weights and relative organ weights of the heart, liver, spleen, lung, kidney, thymus, and ovary or testis were measured. Only the dose of 300 mg/kg of SPE produced a significant reduction (*p* < 0.05) in the lung weight of male rats when compared to that in the control group. However, there was no significant (*p* > 0.05) influence of SPE administration for 28 days on the relative weights of male and female rats (Table 4 and Table 5).

**Figure 4:**
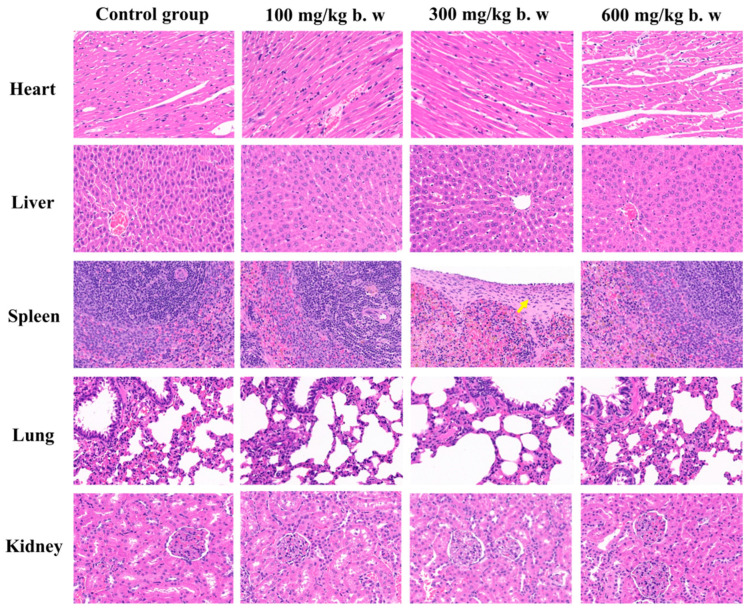
Selected microphotographs (H&E × 400) of heart, liver, spleen, lungs, and kidneys in female rats from the respective groups. Yellow arrow indicates the thickening of the spleen capsule.

The histopathological changes were also examined in the heart, liver, spleen, lungs, and kidneys of both untreated and treated animals. No pathological lesions or inflammatory infiltration indicating abnormalities or toxicity were detected in any of the organs from the control group or the groups treated with 100, 300, and 600 mg/kg bw of SPE, except one slight thickening of the spleen capsule in the 300 mg/kg group (Figure 4). The present histological findings further confirm the safety of SPE, which did not pose health risks related to acute or sub-acute toxicity.

## 3. Materials and Methods

### 3.1. Material and Reagents

Amygdalin, ascorbic acid, 1,1-diphenyl-2-picrylhydrazide (DPPH), (2,2-azino-bis(3-ethylbenzothiazoline-6-sulfonate)) (ABTS), and HPLC-grade methanol were all purchased from Sigma-Aldrich (St. Louis, MO, USA). Water was purified using a water purification system (Chengdu, China). Petroleum ether, ethanol, and the other reagents were commercially available and of analytical grade.

### 3.2. Ethanol Extraction from Semen Persicae

Semen persicae was collected from Gansu Province, China. For this experiment, dried powder of semen persicae (100 g) was treated with 500 mL petroleum ether (at 60 °C for 6 h) for degreasing three times. The crud extraction was dried at 105 °C and subjected to extraction with 85% ethanol at reflux for 2 h. The extract was then filtered, concentrated in a rotary evaporator, and dried under reduced pressure (90 Kpa) in an oven at 45 °C. The obtained dried powder was stored in a refrigerator (2–8 °C) until the beginning of testing.

### 3.3. Determination of Amygdalin in SPE

The HPLC analysis was carried out on Agilent 1260 Infinity Binary Pump system (Santa Clara, CA, USA) equipped with an auto-sampler and a UV detector. All instrument parts were automatically controlled by OpenLAB CDS software (Santa Clara, CA, USA; Revision: C. 01. 07) supplied from Agilent Corporation (Santa Clara, CA, USA). According to the reported methods [26], commercial standards of D-amygdalin (5 mg) were dissolved in 70% MeOH/H_2_O solution (*v*/*v*) (5 mL) and used as standard stock solutions. Working standard solutions were further prepared by appropriate dilution of the stock standard solutions with the same MeOH/H_2_O solution to generate calibration curves. For the determination of amygdalin in SPE, 300 mg of extract was weighed accurately and dissolved to 70% MeOH/H_2_O solution (*v*/*v*) as the test sample. Prior to use, all sample solutions were filtered through a 0.22 µm syringe filter and degassed using an ultrasonic bath for 2 min. After injection of 20 μL of the aforementioned sample solution, chromatographic separation was carried out on a Zorbax SB-C18 (250 mm × 4.6 mm × 5 μm) analytical column (Santa Clara, CA, USA), with the column temperature maintained at 30 °C. The isocratic elution with a mobile phase of methanol-ultrapure water (20:80, *v*/*v*) was pumped at a flow rate of 1.0 mL/min throughout the HPLC process. Chromatograms were monitored at 210 nm with a runtime of 35 min.

### 3.4. Animals

Fifty-two adult specific pathogen-free (SPF) Sprague–Dawley rats (6–8 weeks old, 190 ± 10 g body weight for acute oral toxicity study and 170 ± 30 g body weight for subchronic toxicity study) were purchased from the Laboratory Animal Center of Lanzhou University, and the animal studies were carried out in accordance with the ethical principles of animal research and approved by the Ethics Committee of Laboratory Animal Center of Lanzhou University (No. SCXK2023-0005). Animals were kept in clean, stainless steel cages (2–3 rats per cage) with free access to food (SLACOM Inc., Shanghai, China) and water under 23 °C conditions, with a constant 12 h light–dark cycle. After being acclimatized for at least 7 days, the animals were used for the experiments, which were conducted between 08:30 AM and 17:30 PM in compliance with the ARRIVE guidelines [27].

### 3.5. DPPH Free Radical Scavenging Assay

The capacity of SPE to scavenge DPPH free radicals was assessed according to the reported method [28] with minor modifications. Briefly, 100 μL SPE and ascorbic acid (positive control) aqueous solutions (2, 1, 0.5, 0.25, 0.125, 0.0625 and 0.03125 mg/mL) were mixed thoroughly to 200 μL ethanolic 0.2 mM DPPH solution, respectively, in 96-well plates. These prepared solutions were kept for 30 min in a dark environment at room temperature, followed by measuring the absorbance at 517 nm using an ultraviolet visible spectrophotometer. The DPPH scavenging rate (%) was determined as: scavenging rate (%) = [1 − (As − A_1_)/A_0_] × 100, where As represents the absorbance for the sample with DPPH, A_1_ represents the absorbance for the sample without DPPH, and A_0_ represents the absorbance measured for the DPPH solution without a sample.

### 3.6. Hydroxyl Radical Scavenging Assay

The hydroxyl free radical scavenging of SPE has been assessed by previous reports [6,29]. In brief, 50 μL SPE and ascorbic acid (positive control) aqueous solutions (the same concentrations as that in DPPH free radical scavenging assay) were thoroughly mixed separately with 50 μL FeSO_4_ solution (9 mM) and 50 μL ml ethanolic salicylic acid solution (9 mM). The prepared mixture solutions were added to 50 μL H_2_O_2_ (3.8 mM) and shaken well, followed by completion of the reaction in a water bath at 37 °C for 30 min. The absorbances were estimated using a UV-VIS spectrophotometer at a 510 nm wavelength, and the hydroxyl radical scavenging rate (%) was estimated as: scavenging rate (%) = [1 − (As − A_1_)/A_0_] × 100, where As represents the absorbance for the sample with H_2_O_2_, A_1_ represents the absorbance for the sample without H_2_O_2_, and A_0_ represents the absorbance for the H_2_O_2_ without a sample.

### 3.7. ABTS Radical Cation Decolorization Assay

The antioxidant activity of the SPE was also studied using the ABTS radical cation decolorization assay according to previous reports [30]. The ABTS radical cation (ABTS^+^) was produced by reacting ABTS solution (7 mM concentration in deionized water) with 2.45 mM potassium persulfate (K_2_S_2_O_8_) and was kept in the dark at room temperature for 12–16 h. Then, ABTS^+^ solution was diluted in PBS buffer (pH = 6.6) to an absorbance of 0.75 ± 0.02 at 734 nm. After adding 100 µL of SPE and ascorbic acid (positive control) aqueous solutions (2, 1, 0.5, 0.25, 0.125, 0.0625 and 0.03125 mg/mL) to 3.9 mL of ABTS^+^ solution, the mixture was mixed thoroughly at 30 °C for 10 min, and the absorbance reading was taken. The same determinations were carried out in triplicate. The percentage of inhibition of ABTS^+^ was calculated using the same formula as that in the DPPH free radical scavenging assay.

### 3.8. Fe^2+^ Chelating Activity

The Fe^2+^-chelating abilities of SPE or EDTA (positive control) were determined to resemble the previous reports [4,31]. In this assay, 100 μL SPE and EDTA (positive control) at different concentrations (2, 1, 0.5, 0.25, 0.125, 0.0625 and 0.03125 mg/mL) were added to a solution of 2 mM FeCl_2_ (5 μL). The reaction was initiated by the addition of 5 mM ferrozine (20 μL), and the mixture was shaken vigorously and kept for 10 min at room temperature. After the addition of 75 μL distilled water, the absorbance was measured at 560 nm. The percentage inhibition of ferrozine–Fe^2+^ complex formation (%) was determined using the following formula: chelating activity (%) = [1 − (As − A_1_)/A_0_] × 100, where As represents the absorbance of the sample with ferrozine, A_1_ represents the absorbance of the sample without ferrozine, and A_0_ represents the absorbance of the control which contained ferrozine.

### 3.9. Cytotoxicity Assay

Cytotoxicity of SPE was performed against RAW 264.7 and HepG2 cells following the same protocol as earlier reports [32,33], with some modifications. Cells were counted and inoculated into 96-well plates with approximately 6 × 10^3^ cells/well density, then for incubated for 4 h at 37 °C and 5% CO_2_. Various concentrations of SPE in 100 μL fresh medium (16.125, 31.25, 62.5, 125, 250, 500, 1000, and 2000 mg/mL) were added directly into the plates and co-incubated for 24 h at 37 °C with 5% CO_2_. After the medium was removed, 100 μL of CCK-8 in medium (10%) solution was added in darkness for incubation at 37 °C for 4 h. The mediums were then tested at 450 nm using an enzyme marker (BioTek Instruments Inc., Winooski, VT, USA). The same procedure was repeated three times. The percentages of cell viability were calculated by the following formula: cell viability (%) = [A_sample_ − A_negative_]/[A_positve_ − A_negative_] × 100%.

### 3.10. Acute Oral Toxicity Study

A single-dose acute oral toxicity study of SPE was performed according to the OECD Test Guideline 423 bulletin [34]. We initially selected 2000 mg/kg of SPE dissolved in vehicle (Tween-80: DMSO: physiological saline = 4:4:2) as the start dose for the first round of treatment. Animals in the control group (*n* = 3) only received 2 mL of the vehicle. Behavioral changes and mortality were observed consciously for the first 4 h and once a day for 14 consecutive days. Because no animals died during the first 7 days after treatment, the second round of treatment was performed with the same procedure on three additional female rats. At the end of the observation period (14 days), all the surviving animals were euthanized by means of deep anesthesia with 70 mg/kg of ketamine hydrochloride (i.p.), and their hearts, livers, spleens, lungs, kidneys, uterus, and ovaries were individually observed for overt pathology and removed for relative weight calculation (organ/bw × 100).

### 3.11. Repeated Dose 28-Day Oral Toxicity

We conducted an evaluation of the short-term exposure of SPE following the procedure of the OECD Guideline 407 bulletin [35] using 40 Sprague–Dawley rats (20 males, 20 females). Rats were separated into four treatment groups (five males and five females per group) and were orally administered SPE that was dissolved in vehicle, the same as that in acute oral toxicity study. Doses were selected based on the LD_50_ value obtained from our acute oral toxicity, and 100, 300, and 600 mg/kg of body weight were set as the low-, middle-, and high-dose groups, respectively. Rats in the control group were only administered the vehicle (2 mL/kg). The SPE and vehicle were administered daily orally at the same time (9:00 AM) for 28 days. Monitoring of clinical changes and mortality was performed on a daily basis. Body weight and food intake were assessed on a weekly basis. On the 29th day, the animals were euthanized with diethyl ether, and blood samples were collected via cardiac puncture in test tubes either containing or without EDTA for evaluation of the hematological and biochemical parameters, respectively. Vital organs such as the heart, liver, spleen, lung, and kidney from female rats were carefully dissected for the determination of relative organ weights and histopathological examination.

### 3.12. Statistical Analysis

The results are expressed as mean ± standard deviation (SD). Differences between groups were determined by a one-way analysis of variance (ANOVA) followed by Dunnett’s post hoc tests using IBM SPSS Statistics for Windows (Armonk, NY, USA), version 24.0 [36]. Statistically significant differences were defined as *p* < 0.05, and extremely significant differences were defined as *p* < 0.01.

## 4. Discussion

Many plant extracts or respective formulations have been revealed to have the function of antioxidant activity. For example, polysaccharides, which are well known in many plants and fungi, have strong antioxidant activities [37,38]. Lycopene, a carotenoid which is abundant in mature red plant fruits, has been found to display good antioxidant capacity with strong scavenging ability on DPPH and ABTS free radicals [39]. Semen persicae has been used as a traditional Chinese medicine (TCM) formulation with a wide range of pharmacological effects [7,10,11,21]. However, there is limited information about the effects of semen persicae extracted by alcohol on antioxidant activity.

Amygdalin is highly concentrated in semen persicae with various biological activities, and therefore, it is used as a chemical indicator to control the quality of this TCM formulation [20]. Because of the poor stability of amygdalin, for example, in water, it will be decomposed into benzaldehyde, HCN, and glucose by emulsin (a hydrolysis enzyme in semen persicae) [40]. Its content is largely influenced by the processing method. Using our extract protocol, we obtained 4.78% of amygdalin determined by reversed-phase separation, with 20% methanol as a mobile phase after extraction with 85% ethanol.

The antioxidant activities of SPE were evaluated by determining the DPPH, the hydroxyl and ABTS^+^ scavenging effect, and the Fe^2+^-chelating activity. The DPPH free radical scavenging assessment is widely employed to assess the antioxidant property of many bioactive compounds [41]. In addition, hydroxyl radicals are highly reactive with most biological macromolecules, resulting in human health damage. Thus, eradicating avoidable hydroxyl radicals is another valid method to evaluate the antioxidant property of an agent [42,43]. For the antioxidant capacity test of plant extracts, the ABTS assay is particularly interesting because the wavelength absorption at 734 nm eliminates color interference, which requires relatively standard equipment and delivers fast and reproducible results [30,44]. In our research, significant increases in the DPPH, hydroxyl, and ABTS^+^ scavenging rates were observed with increasing SPE concentrations, indicating that SPE had a clear antioxidant effect. However, SPE displayed low chelating activity, with estimated an 9.95 mg/mL IC_50_ value. This result allows us to conclude that SPE might be slightly beneficial in protecting against oxidative damage by inhibiting the production of reactive oxygen species and lipid peroxidation [4].

Although antioxidant capacity is one of the biological functions of amygdalin, very few reports have found that the antioxidant activity of SPE is related to its content [45,46]. However, the antioxidant activity of a plant extract can probably be attributed to high levels of phenolic compounds that bear the ability to act as hydrogen or electron donors and scavenge free radicals [46,47].

It is well known that amygdalin is one of the most important active ingredients of semen persicae [11,19]. Unfortunately, this compound can ultimately be transformed to toxic hydrogen cyanide in animals [48,49]. In HPLC analysis, ethanol extract of semen persicae contained 4.94% amygdalin. Therefore, an evaluation of the safety of this extract is urgently required.

Cytotoxicity evaluation of SPE was completed with a maximum concentration up to 2000 μg/mL because of the slightly water-soluble property. Even at this concentration, SPE displayed negligible cytotoxicity, with 92.45% cell viabilities of HepG2 and no significantly (*p* ≥ 0.05) reduced cell viabilities of RAW 264.7.

Acute toxic assessment, which usually serves as a preliminary step in the screening of the pharmacological activity of natural products, may provide initial data on the toxic mode of action, the basis for identification and classification, and the safety level in in vivo studies [50]. Therefore, we evaluated the in vivo acute toxicity of SPE using OECD guideline 423. This method can measure a drug’s rough LD_50_ with fewer experimental animals. We initially selected 2000 mg/kg of SPE as a starting point. The results indicate that no mortality or signs of toxicity at the macroscopic examination were found, indicating that its LD_50_ value should be more than 2000 mg/kg body weight according to OECD Guidelines 423 [32]. This result was consistent with the LD_50_ of pre-brewed Armeniacae semen aqueous extracts in female and male rats [25].

The daily administration of SPE at doses up to 600 mg/kg for 28 days did not cause mortality or clinical toxicity signs, nor did it induce changes in organ relative weight, which may be indicative of edema, atrophy, or hypertrophy of the organs [51]. However, a significant decrease in lung weight was found in the 300 mg/kg group of male rats when compared to the control group. Considering it was not a dose-dependent relationship, and considering the histopathological results, we can assume that the changes were not related to organ toxicity caused by 300 mg/kg SPE.

Repeated administration of SPE 28 days caused minor but statistically significant hematological changes in MPV (*p* < 0.05 or *p* < 0.01), which occurred in both male and female rats. However, these minor changes, although statistically significant, were assumed to be toxicologically irrelevant because they remained within the normal range [52], revealing that SPE did not demonstrate any haematotoxicity effects. The serum biochemical parameter creatinine, which is considered as a sensitive biomarker of renal pathologies [53,54], was significantly increased after 28 days of treatment. Although this parameter was significantly altered, it was also within the normal range [53,55]. Furthermore, the biochemical parameter of urea (other marker of renal function) and the histopathological analysis of the kidneys showed no abnormal signs, supporting the notion that the significantly increased creatinine was not associated with the toxicology of SPE.

Studies have proven that the generation of toxicity of semen persicae is mainly related to overdosed amygdalin [20,21]. Amygdalin is non-toxic itself, but is ultimately transformed into benzaldehyde and hydrogen cyanide; the latter can inhibit cell respiration and bind to cytochrome oxidase, which causes cell hypoxia and lactic acidosis [56,57]. The 2000 mg/kg bw oral dose of SPE in our acute toxicity was equal to a 98.8 mg/kg bw oral dose of amygdalin (content in SPE was 4.94%), which was notably lower than the reported mean lethal dose (LD_50_ = 880 mg/kg bw) of orally administrated amygdalin for rats [25,57].

## 5. Conclusions

This study explored the antioxidant activity and toxicity profiles of the ethanolic extract of semen persicae (SPE). This extraction demonstrated better antioxidant activities in DPPH, hydroxyl, and ABTS^+^ scavenging ability, with scavenging rates up to 51.78%, 55.47%, and 57.16% at a 2 mg/mL concentration, respectively, but exhibited low Fe^2+^-chelating activity (30.76%) at the same concentration. Cell viabilities revealed negligible cytotoxicity up to 2000 μg/mL. Based on the acute oral toxicity data, the LD_50_ value of SPE may be considered to be more than 2000 mg/kg body weight. According to the data from the subchronic toxicity study, we finally concluded that the daily administration of SPE at doses of 100, 300, and 600 mg/kg for 28 days did not cause mortality or induce adverse effects in female or male rats. These findings provide a theoretical foundation for the therapeutic applications of SPE and present a promising avenue for the development of effective antioxidant agents in the pharmaceutical industry.

## Figures and Tables

**Table 1:** Absolute and relative organ weights of female rats in acute oral toxicity study.

Parameters	Control Group	First Round Treatment	Second Round Treatment	*p*_1_ ^a^	*p*_2_ ^b^
Body weights (g)					
0 day	193.67 ± 2.45	191.40 ± 1.28	191.33 ± 2.42	-	-
14 days	218.93 ± 2.40	216.47 ± 3.16	215.43 ± 4.45	-	-
Absolute organ weight (g)					
Liver	9.0 ± 0.22	8.04 ± 0.53	8.82 ± 0.91	0.205	0.914
Kidneys	1.72 ± 0.11	1.63 ± 0.18	1.60 ± 0.13	0.744	0.581
Spleen	0.57 ± 0.04	0.53 ± 0.02	0.53 ± 0.05	0.395	0.558
Heart	0.76 ± 0.08	0.74 ± 0.05	0.82 ± 0.09	0.959	0.580
Lungs	1.27 ± 0.16	1.34 ± 0.10	1.62 ± 0.48	0.951	0.372
Uterus + ovaries	0.88 ± 0.06	0.76 ± 0.04	1.00 ± 0.20	0.509	0.508
Relative organ weight (%)					
Liver	4.12 ± 0.07	3.71 ± 0.20	4.09 ± 0.34	0.152	0.983
Kidneys	0.78 ± 0.04	0.75 ± 0.07	0.74 ± 0.05	0.785	0.617
Spleen	0.26 ± 0.02	0.24 ± 0.01	0.25 ± 0.02	0.443	0.726
Heart	0.35 ± 0.04	0.34 ± 0.03	0.38 ± 0.05	0.974	0.556
Lungs	0.58 ± 0.02	0.62 ± 0.05	0.76 ± 0.19	0.902	0.233
Uterus + ovaries	0.40 ± 0.03	0.35 ± 0.02	0.46 ± 0.09	0.562	0.384

Note: ^a^ *p* values of significant differences between the control group and the first-round treatment; ^b^ *p* values of significant differences between the control group and the second-round treatment.

**Table 2:** Effects of 28-day repeated dose of SPE on hematological parameters of Sprague–Dawley rats (mean ± SD).

Parameters	Control	100 mg/kg bw	300 mg/kg bw	600 mg/kg bw
Female				
WBC (10^9^/L)	5.42 ± 0.95	5.77 ± 1.46	6.03 ± 1.12	6.02 ± 1.09
NEU (10^9^/L)	0.70 ± 0.13	0.80 ± 0.17	0.78 ± 0.19	0.64 ± 0.15
NEP (%)	12.86 ± 2.22	12.90 ± 1.94	11.98 ± 2.16	11.68 ± 2.34
RBC (10^12^/L)	8.26 ± 0.58	8.27 ± 0.70	7.79 ± 0.61	8.07 ± 0.71
HGB (g/dL)	15.90 ± 1.19	15.38 ± 1.34	15.68 ± 1.32	14.84 ± 0.97
HCT (%)	49.33 ± 4.54	48.19 ± 5.42	52.85 ± 3.86	50.04 ± 3.60
MCV (fL)	58.77 ± 3.27	60.10 ± 2.81	59.20 ± 4.45	58.46 ± 4.58
MCH (pg)	19.06 ± 1.05	18.98 ± 1.51	19.04 ± 0.87	18.68 ± 0.96
MCHC (g/dL)	31.22 ± 1.71	31.12 ± 2.21	30.72 ± 2.69	31.96 ± 2.36
PLT (10^9^/L)	669.4 ± 70.6	645.1 ± 105.8	699.8 ± 121.2	724.2 ± 112.8
MPV (fL)	6.50 ± 0.16	6.72 ± 0.17	7.04 ± 0.27 *	7.16 ± 0.30 **
Male				
WBC (10^9^/L)	5.86 ± 0.99	5.93 ± 0.95	5.82 ± 1.77	6.44 ± 1.64
NEU (10^9^/L)	0.71 ± 0.13	0.75 ± 0.15	0.75 ± 0.13	0.70 ± 0.07
NEP (%)	11.78 ± 2.33	12.64 ± 1.83	12.92 ± 2.31	12.04 ± 2.36
RBC (10^12^/L)	8.42 ± 0.66	8.16 ± 0.90	8.49 ± 0.89	8.45 ± 0.76
HGB (g/dL)	15.92 ± 1.12	16.10 ± 1.10	16.24 ± 1.25	16.02 ± 0.92
HCT (%)	49.81 ± 6.24	51.54 ± 2.15	52.00 ± 3.72	50.82 ± 2.65
MCV (fL)	60.10 ± 3.61	60.77 ± 2.67	60.80 ± 2.48	59.77 ± 2.06
MCH (pg)	19.02 ± 1.14	18.92 ± 0.94	19.35 ± 1.22	19.37 ± 1.32
MCHC (g/dL)	30.76 ± 1.78	31.82 ± 2.32	32.04 ± 1.53	31.06 ± 1.58
PLT (10^9^/L)	657.6 ± 85.1	593.6 ± 80.2	751.8 ± 176.2	628.2 ± 84.7
MPV (fL)	6.66 ± 0.21	6.76 ± 0.22	6.96 ± 0.34	7.26 ± 0.22 *

Abbreviations: WBC, white blood cells; NEU, neutrophil; NEP, percent of neutrophil; RBC, red blood cells; HGB, hemoglobin; HCT, hematocrit; MCV, mean corpuscular volume; MCH, mean corpuscular hemoglobin; MCHC, mean corpuscular hemoglobin concentration; PLT, platelets; MPV, mean platelet volume. Values represent means ± SD. * *p* < 0.05 vs. control group (0 mg/kg bw); ** *p* < 0.05 vs. control group (0 mg/kg bw).

**Table 3:** Biochemical parameters of rats sacrificed on day 28 of 28-day feeding test (mean ± SD).

Female				
ALT (U/L)	37.42 ± 5.87	41.34 ± 8.21	36.57 ± 5.85	37.80 ± 7.17
AST (U/L)	132.00 ± 9.31	129.08 ± 9.98	125.10 ± 13.21	133.54 ± 7.41
TC (mmol/L)	1.75 ± 0.25	1.78 ± 0.18	1.82 ± 0.20	1.73 ± 0.14
Urea (mmol/L)	9.53 ± 0.72	9.74 ± 0. 97	9.44 ± 0.53	9.92 ± 0.88
Crea (μmol/L)	41.48 ± 6.61	46.68 ± 4.53	54.78 ± 7.01 *	46.80 ± 7.17
ALP (U/L)	209.30 ± 50.43	197.68 ± 56.56	185.58 ± 62.74	194.90 ± 67.64
ALB (g/L)	36.96 ± 2.67	35.48 ± 2.85	36.00 ± 3.97	35.80 ± 4.57
Male				
ALT (U/L)	43.02 ± 4.88	46.24 ± 7.54	49.06 ± 6.19	47.26 ± 7.31
AST (U/L)	138.34 ± 8.73	135.08 ± 9.48	140.80 ± 5.71	139.74 ± 9.87
TC (mmol/L)	1.71 ± 0.12	1.80 ± 0.09	1.77 ± 0.16	1.94 ± 0.14
Urea (mmol/L)	9.61 ± 0.93	9.44 ± 0.47	9.57 ± 0.72	9.57 ± 0.85
Crea (μmol/L)	41.48 ± 9.19	40.34 ± 7.10	45.76 ± 9.01	41.48 ± 8.85
ALP (U/L)	225.06 ± 34.37	195.78 ± 47.93	213.88 ± 53.80	223.66 ± 48.25
ALB (g/L)	34.32 ± 3.70	37.16 ± 3.62	34.80 ± 3.17	35.42 ± 3.08

Abbreviations: ALT, alanine transaminase; AST, aspartate transaminase; TC, total cholesterol; Crea, creatinine; ALP, alkaline phosphatase; ALB, albumin. Values represent means ± SD. * *p* < 0.05 vs. control group (0 mg/kg bw).

**Table 4:** Absolute and relative organ weights of female rats in 28-day repeated-dose toxicity study.

Parameters	Control Group	100 mg/kg bw	300 mg/kg bw	600 mg/kg bw
Body weight (g)				
0 days	165.18 ± 8.32	159.66 ± 11.67	158.80 ± 7.45	161.81 ± 9.05
28 days	208.84 ± 7.24	211.68 ± 8.33	207.93 ± 7.89	205.03 ± 9.56
Absolute organ weight (g)				
Heart	0.85 ± 0.12	0.83 ± 0.11	0.91 ± 0.06	0.88 ± 0.08
Liver	7.75 ± 1.07	7.93 ± 0.69	8.09 ± 1.01	6.96 ± 1.16
Spleen	0.49 ± 0.07	0.48 ± 0.06	0.56 ± 0.08	0.55 ± 0.11
Lungs	1.23 ± 0.13	1.14 ± 0.13	1.21 ± 0.10	1.27 ± 0.20
Kidneys	1.57 ± 0.07	1.65 ± 0.06	1.61 ± 0.09	1.60 ± 0.05
Thymus	0.50 ± 0.11	0.52 ± 0.06	0.49 ± 0.04	0.47 ± 0.05
Ovaries	0.13 ± 0.03	0.14 ± 0.03	0.15 ± 0.03	0.15 ± 0.02
Organ-to-body-weight ratio (%)				
Heart	0.41 ± 0.06	0.40 ± 0.06	0.44 ± 0.02	0.43 ± 0.02
Liver	3.71 ± 0.47	3.76 ± 0.43	3.90 ± 0.57	3.38 ± 0.49
Spleen	0.23 ± 0.03	0.23 ± 0.02	0.27 ± 0.03	0.27 ± 0.06
Lungs	0.59 ± 0.08	0.54 ± 0.06	0.58 ± 0.04	0.62 ± 0.09
Kidneys	0.75 ± 0.01	0.78 ± 0.01	0.77 ± 0.02	0.78 ± 0.04
Thymus	0.24 ± 0.05	0.25 ± 0.02	0.24 ± 0.01	0.23 ± 0.02
Ovaries	0.06 ± 0.01	0.07 ± 0.01	0.07 ± 0.01	0.08 ± 0.01

Values are mean ± SD for 10 rats in each group.

**Table 5:** Absolute and relative organ weights of male rats in 28-day repeated-dose toxicity study.

Parameters	Control Group	100 mg/kg bw	300 mg/kg bw	600 mg/kg bw
Body weight (g)				
0 days	192.04 ± 8.83	188.42 ± 13.86	193.10 ± 10.35	190.35 ± 10.62
28 days	257.15 ± 10.82	254.43 ± 10.27	251.73 ± 7.21	249.70 ± 11.66
Absolute organ weight (g)				
Heart	0.94 ± 0.10	0.92 ± 0.12	0.97 ± 0.14	0.98 ± 0.10
Liver	9.24 ± 1.56	9.45 ± 1.44	9.19 ± 1.32	9.33 ± 1.17
Spleen	0.58 ± 0.07	0.62 ± 0.07	0.65 ± 0.09	0.62 ± 0.06
Lung	1.50 ± 0.09	1.33 ± 0.16	1.32 ± 0.08 *	1.34 ± 0.11
Kidney	2.12 ± 0.14	2.04 ± 0.18	2.10 ± 0.23	1.63 ± 0.37
Thymus	0.57 ± 0.11	0.50 ± 0.13	0.56 ± 0.13	0.59 ± 0.15
Testis	3.06 ± 0.25	2.92 ± 0.25	2.87 ± 0.18	3.16 ± 0.26
relative organ weight				
Heart	0.37 ± 0.03	0.36 ± 0.04	0.39 ± 0.05	0.39 ± 0.06
Liver	3.58 ± 0.47	3.72 ± 0.61	3.64 ± 0.45	3.73 ± 0.31
Spleen	0.23 ± 0.03	0.25 ± 0.02	0.26 ± 0.03	0.25 ± 0.02
Lungs	0.58 ± 0.01	0.52 ± 0.05	0.52 ± 0.05	0.54 ± 0.04
Kidneys	0.83 ± 0.05	0.80 ± 0.05	0.83 ± 0.07	0.65 ± 0.15
Thymus	0.22 ± 0.05	0.20 ± 0.05	0.22 ± 0.05	0.24 ± 0.06
Testis	1.19 ± 0.06	1.15 ± 0.06	1.14 ± 0.08	1.27 ± 0.09

Values are mean ± SD for 10 rats in each group. * Statistically significant difference compared to control (*p* < 0.05).

## Data Availability

Data will be made available upon request.

## Supplementary Materials

The following supporting information can be downloaded at: https://www.mdpi.com/article/10.3390/ijms25168580/s1.
