# Supplementary material for: Antioxidant Activity and Preclinical Safety of Semen persicae Extract
Source: Int J Mol Sci. 2024 Aug 6;25(16):8580. doi: 10.3390/ijms25168580 (PMC11354697; doi:10.3390/ijms25168580)
Supplement: Supplementary file 1 [file ijms-25-08580-s001.zip › ijms-3131658-supplementary.pdf]

Effects on antioxidant activity and preclinical safety of *Semen persicae* extract

Jing Yang, Yingying Song, Yu Liu, Rui Zhang, Liqiong Jin*

Gansu Analysis and Research Center, Lanzhou, 730000, China

Corresponding author: Tel.: +86 931-8873565; fax: +86 931-8631006. E-mail address: jew168@sina.com (Liqiong Jin).

Author: Tel. +86 931-8631006; fax: +86 931-8631006. E-mail address: phoenixdudu@126.com (Jing Yang).

Address: Gansu Analysis and Research Center, No. 225, Dingxi south road, Chengguan District, Lanzhou, 730000, P.R. China.

Supplemental Data

Table of Contents

Table S1. Body weight of female rats in acute toxicity study 1

Figure S1. Food consumptions of female and male rats administered with SPT in subacute toxicity study..........3

Figure S2. Body weights of female and male rats fed with SPT for 28 days...4

Table S1. Body weight of female rats in acute toxicity study

Treatment (dose)	Rat No.	Body weight (g)				
		Initial	Day 7	Weight change at day 7	Day 14	Weight change at day 14
First round (2000 mg/kg b.wt.)	1	192.8	209.1	16.3	220.1	27.3
	2	191.1	204.8	13.7	214.9	23.8
	3	190.3	207.5	17.2	214.4	24.1
Second round (2000 mg/kg b.wt.)	1	193.2	206.0	12.8	218.2	25.0
	2	188.6	204.9	16.3	210.3	21.7
	3	192.2	207.5	15.3	217.8	25.6
Control group	1	196.2	210.7	14.5	218.8	22.6
	2	191.3	208.2	16.9	216.6	25.3
	3	193.5	210.0	16.5	221.4	27.9

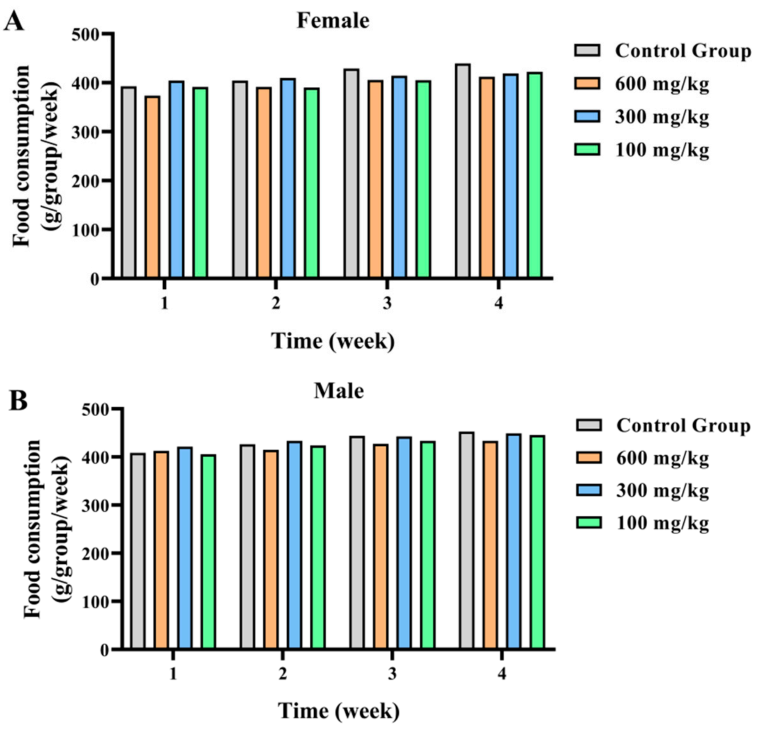

Figure S1. Food consumptions of female (A) and male (B) rats administered with SPT in subacute toxicity study.

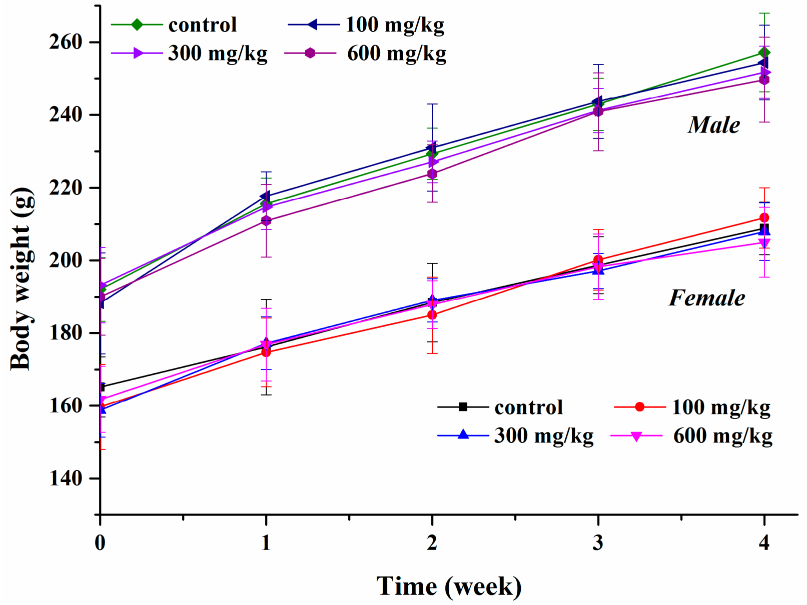

Figure S2. Body weights of female and male rats fed with SPT for 28 days
